# Parotitis after Operations

**Published:** 1908-09-19

**Authors:** 


### PAROTITIS AFTER OPERATIONS.

The parotid gland on the one hand, and the ovaries or testicles upon the other, are apt to be thought to have some relationship to one another as regards disease, and thus it came about that for a long time the teaching was that, of all operations, that most likely to be followed by parotitis was ovariotomy. Perhaps there were two chief things which predisposed to the coupling together of parotid and ovary or parotid and testicle—namely, first, the fact that mumps or specific parotitis is very apt to cause orchitis or ovaritis, either at the same time as the parotitis or as a sequel to it, or even as an alternative to it; and, secondly, the fact that certain peculiar tumours of the testicle or ovary containing cartilage and so forth were once thought to be of the same nature as certain mixed tumours of the parotid glands, in which cartilage may also be found. There are still some who hold that ovariotomy is more apt to be succeeded by parotitis than any other operation is, but an investigation of the matter shows, first, that no serious surgical operation is quite free from the chance of being complicated by parotitis during recovery; secondly, that the more serious the operation—or, at any rate, the more serious the patient's general condition—the more likely is the parotitis to develop; thirdly, that a precisely similar parotitis may arise in cases in which no operation has been performed at all, but in which the patient has been ill in bed in an asthenic state for a long time, as in typhoid fever for example; fourthly, that parotitis as a complication either of typhoid fever or of convalescence after a serious operation has become very much rarer than it used to be, and that the greater the care taken to keep the patient's mouth clean the less the likelihood of parotitis developing. From all this it would seem that the operation is only an indirect cause of the parotitis, and that there is little reason to suppose that the fact that it has been performed upon the ovary will predispose to parotitis in any specific way. It has been asserted by some observers that the cleanliness or otherwise of the mouth has nothing to do with the parotitis, and that the latter is metastatic—that is to say, due to infection by the blood-stream and not by Stenson's duct. This may be the case sometimes, but it certainly is not so always. In some cases a different organism has been found in the inflamed parotid gland to that which is causing the inflammation at the site of operation—bacillus coli communis in the vermiform appendix, for example, and staphylococcus in the parotid gland. The practical lesson which a study of post-operative parotitis teaches is that the greater the care taken with the patient's mouth and teeth the less the likelihood of the complication.

